# Ultradian rhythms of activity in a wild subterranean rodent

**DOI:** 10.1098/rsbl.2024.0401

**Published:** 2024-10-23

**Authors:** Kyle T. Finn, Otto Brede, Nigel C. Bennett, Markus Zöttl

**Affiliations:** ^1^ Department of Zoology and Entomology, University of Pretoria, Pretoria, South Africa; ^2^ Ecology and Evolution in Microbial Model Systems, Department of Biology and Environmental Science, Linnaeus University, Kalmar, Sweden

**Keywords:** locomotor activity, behaviour, circadian clock, mole-rat, polyphasic activity

## Abstract

Many animals adapt their activity patterns to the best environmental conditions using daily rhythms. African mole-rats are among the mammals that have become models for studying how these rhythms can be entrained by light or temperature in experimental laboratory studies. However, it is unclear whether they exhibit similar circadian rhythms in their natural lightless, subterranean environment. In this study, we used biologging to investigate the activity rhythms of wild, highveld mole-rats. We show that their activity cycle exhibited an ultradian rhythm with a length between 4 and 8 h. On an individual level, mole-rats displayed about five activity bouts per day, occurring at various times during the day and night. On a population level, activity peaked in the afternoon, coinciding with the peak in ambient temperature. Our research suggests that wild subterranean mammals, which experience reduced environmental variation, are unlikely to show clear circadian rhythmicity in activity patterns. Instead, activity periods are distributed over several bouts throughout the day and night, and activity coincides with the peak in daily temperature. We propose that ultradian rhythms in activity may be more common than previously thought and discuss how physiological processes may generate differences in periodicity between laboratory and wild populations.

## 1. Introduction

The majority of animal species synchronize, or entrain, their biological rhythms to the rising and setting of the sun and thus show a 24 h rhythm of behaviour and physiology [1]. Circadian rhythms have a period length of approximately 24 h, with an internal, or endogenous, clock controlling their expression and will persist under constant conditions (e.g. complete darkness). However, some daily rhythms may only persist in the presence of a light–dark cycle and break down under constant conditions (e.g. lack of a light–dark cycle in Arctic regions [2,3]). Some mammals, particularly those where physiological constraints (e.g. diet, metabolism) render 24 h rhythms suboptimal, show ultradian activity rhythms with multiple cycles of 2–6 h per day (Soricidae [4,5]; Arvicolinae [6–8]). For example, activity rhythms in voles occur in cycles lasting 2–3 h due to their small body mass, metabolic demands and poor nutrient absorption of their grass and forb heavy diet [6]. Some small mammal species may experience both a daily and an ultradian rhythm simultaneously in different biological processes, but individuals who have the controlling mechanisms of their circadian clock removed may continue to experience ultradian rhythms [9,10]. Because cyclicity affects many aspects of the animals’ physiology the mechanisms behind this cyclicity is an important area of biomedical research related to the effects of disrupted circadian rhythms [11,12].

While light serves as the primary entrainer (or zeitgeber) for regulating biological rhythms in most land-living animals, the biological rhythms of organisms in dark habitats and how they maintain their internal clocks remain less understood. Many species inhabiting caves, deep sea environments, and subterranean burrows—where exposure to sunlight is minimal or absent—display circadian rhythms [13,14]. This phenomenon occurs despite the near constant darkness, and the fact that some of these animals lack eyes entirely or have regressed eyes [13]. Indeed, the visual system plays a supportive role in synchronizing (i.e. entraining) the internal clock by detecting changes in light [14–16]. Even infrequent light exposure may be enough to entrain circadian rhythms in subterranean mammals, but temperature changes may affect the onset of activity bouts [17,18].

The subterranean African mole-rats (family: Bathyergidae) are among the mammals dwelling in constant darkness, and they have become models for studying how biological rhythms can be entrained by different cues in the laboratory (reviewed in [19]). African mole-rats spend nearly their entire lives underground, and their burrows are permanently sealed so that they are usually not exposed to sunlight [20,21]. Despite the lack of sunlight exposure and rudimentary eyes, mole-rats still possess circadian rhythms in biological functions and activity under constant conditions of darkness and temperature [14,19]. The light detection aspect of mole-rat vision has been retained as both a means to locate tunnel breaches and to allow for the entrainment of circadian rhythms from light signals [14]. Laboratory studies have further revealed that mole-rats exhibit endogenous circadian rhythms of melatonin secretion [22–25], metabolic rates [26,27], body temperature [26,28–31] and even locomotor activity [24,25,31–35]. These rhythms persist even in constant darkness or stable temperatures under laboratory conditions and can be entrained by either light or ambient temperature cycles [29,30,36,37]. Field studies have also shown that activity is closely linked to fluctuating daily and even seasonal changes in burrow temperatures [38–42]. Yet it remains unclear whether mole-rats exhibit 24 h, ultradian or arrhythmic activity patterns in their natural subterranean habitats.

In this study, we investigated the activity rhythms of highveld mole-rats (*Cryptomys pretoriae*) in their natural habitat. Using acceleration biologging in a wild population, we identified distinct bouts of activity for each individual. Our assessment focused on identifying the length of the activity cycles (i.e. 24 h, ultradian or arrhythmic). We explored the distribution of activity bouts throughout the day, both at the individual and population levels. Finally, we tested whether ambient temperature or soil temperature (at the depth of the nest or foraging tunnels) best predicts mole-rat activity.

## 2. Methods

We fitted biologgers to 23 wild highveld mole-rats from six groups at a nature reserve in South Africa. The loggers recorded body acceleration data for 14 days, and we derived activity patterns of individuals from the recordings. We determined the length of the activity period over a 24 h period using a continuous wavelet transformation and then calculated the mean number of bouts per day and mean activity bout length. We then used a mixed model to determine whether time of activity was affected by soil temperature.

### (a) Capture and housing

We captured wild mole-rat groups during summer at Telperion Nature Reserve in South Africa (−25.715, 28.983) using modified Hickman traps baited with sweet potato [43]. The traps were checked every 2 h from approximately sunrise through to 22.00 and left armed overnight. All individuals were sexed, weighed, measured and assigned a reproductive status when captured. Each group contained only one reproductive female, which was easily identified by prominent nipples and a perforate vagina [21]. The heaviest male within the group was assumed to be the reproductive male [20,21]. All other individuals were considered non-reproductive. During capture, mole-rat family groups were housed together in large plastic crates lined with wood shavings and provided with paper towels for nesting material and sweet potato ad libitum for food.

### (b) Collaring and acceleration data collection

We used 23 individual mole-rats from six groups (mean group size + s.d. = 5.8 ± 3.3, range 2–11) for this study. All groups used in this study were complete, with no individuals evading capture. The mole-rats were fitted with a tri-axial accelerometer device (AXY−5 XS, dimensions 15 × 10 × 4 mm, 1.7–2.1 g, TechnoSmart Ltd, Italy) attached around the neck with plastic-coated steel wire and secured with a small metal clamp. We used devices of varying mass and size to match with appropriately sized mole-rats. The entire collar did not exceed 5% of the body mass of the individual. We set the acceleration devices to record continuously at 25 Hz with a max acceleration of 4*g* (1*g* = 9.82 m s^−2^) on a 8-bit scale. Subsequently, we released the mole-rats back into their burrow. The mole-rats were left in their burrow for at least 2 weeks; then subsequently recaptured, and the collars removed before we released the mole-rats. Two devices failed after 5 days, but the remainder of the devices continued collecting data for over 12 days (mean ± s.d. = 12.96 ± 2.22 days, range: 5.34–14.39 days).

### (c) Data analysis

We removed the first 24 h of acceleration data to avoid any unusual behaviour as a consequence of the recent capture. Next, we obtained the overall dynamic body acceleration (ODBA) for each individual [44]. For each acceleration axis, we calculated a running mean over 2 s windows to obtain a static acceleration value. This static value was subtracted from the corresponding value, and absolute values from all three acceleration axes were summed to obtain ODBA values (|*x* – mean(*x*)| + |*y* – mean(*y*)| + |*z* – mean(*z*)|) [45]. ODBA values can be used as a proxy for periods of activity or inactivity based on a threshold, with values above the threshold being periods of activity and values below being inactive [44]. We then considered an individual active when the ODBA values were > 0.15, and assigned it an activity score of 1. Inactivity was then assigned an activity score of 0. Our threshold value was based on accelerometer data of captive highveld mole-rats fitted with the same accelerometer devices and filmed to determine ODBA values for corresponding behaviours (electronic supplementary material, table S1).

All activity analyses and visualizations were performed in R 4.3.0 [46] using the *rethomics* framework, which includes the packages *behavr*, *ggetho*, *zeitgebr* and *sleepr* [47]. To determine the length of the activity period, we used a continuous wavelet transformation to construct a periodogram of activity using the *periodogram* function in *zeitgebr*. Wavelet periodograms may detect ultradian rhythms better than the more traditional Lomb–Scargle and Fourier methods [48]. To further assist with detecting an ultradian activity period, we set the period of interest between 2 and 32 h (instead of the default 16–32 h). We then used the *find_peaks* function in *zeitgebr* to predict the length of the activity cycle. This function creates a peak at the point of time where the rhythm is statistically highest in likelihood to reset; the higher the peak, the more likely the rhythm will reset after the peak [47].

To assess activity bout duration, we calculated the mean ODBA value in 30 min windows per individual. While wild mole-rats may exhibit activity bouts of approximately 60 min [49], our observations of captive Damaraland mole-rats (*Fukomys damarensis*) indicate a mean minimum activity bout length closer to 30 min. We took the running mean of all 2 s interval ODBA values within a 30 min time period. As above, we then assigned an activity score of 1 to windows if the mean ODBA was > 0.15. Subsequently, we used *bout_analysis* from *sleepr* to calculate the mean hours active per day, the mean number of unique activity bouts per day and the mean bout duration. Means are provided as mean ± 1 s.d.

To assess how soil temperature affects activity patterns in the whole population, we measured soil temperature at our study site using data loggers buried in the soil. We buried data loggers at depths representative for the foraging tunnels (10 cm) and nests (30 cm) of highveld mole-rats [50]. Mole-rats dig the majority of their tunnels within the first 10–20 cm of the soil column because this is where their food grows [39,50]. African mole-rats will retreat to a deeper nest when resting [39]. Thus, if animals were active, we assumed they were active in the upper 10 cm of the soil column. To record ambient temperature, a logger was also placed in the sun at a height of 2 m above the ground. Because our loggers recorded the temperature every 10 min, we re-calculated the mean ODBA in 10 min windows and assigned each value an activity score as mentioned above. To test how time of activity was affected by temperature, we converted time of day into a radian value so that time was a value between 0 and 8π, and then built three four-phase wave models in a generalized linear mixed model using the interaction of temperature (either ambient temperature, 10 cm soil temperature, or 30 cm soil temperature) and time as predictors. The four-phase wave model breaks the 24 h day into four periods of 6 h, and then uses a sinus/cosinus function that allows the peaks and the positions of the wave function to vary with the respective temperature. The models used the *glmmTMB* package [51], and animal identity was included as a random factor. To assess what temperature best predicted mole-rat activity among the three models, we compared AIC and marginal *R*^2^ values.

## 3. Results

**Figure 1.**
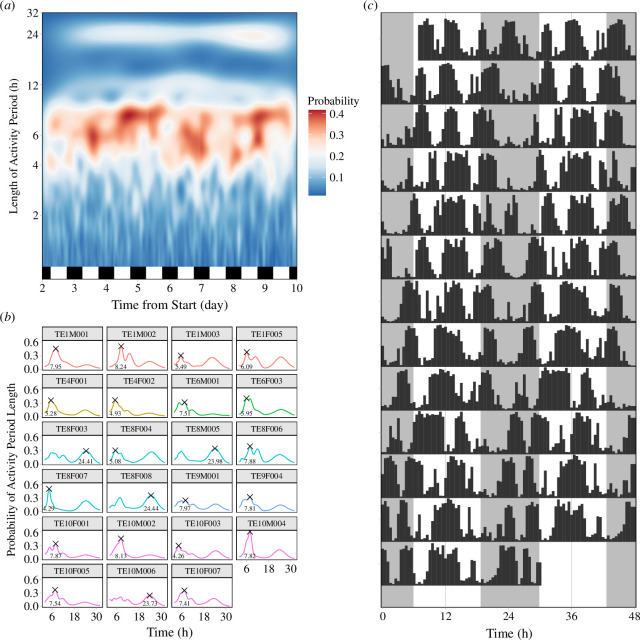
Daily ultradian activity cycles in wild mole-rats. (*a*) A heat map (spectrogram) from the continuous wavelet analysis showing the variation of period length over time for all individuals combined. The colours represent the probability of the period length, red indicates a higher probability. Data from days 2 to 9 are shown. The light–dark cycle is represented as a white and black bar at the bottom. (*b*) The length of the activity period for each individual was predicted using a continuous wavelet transformation periodogram. The X represents the duration of the most likely activity period. Group identity is represented with different colours. (*c*) Example actogram of an individual (TE10F007) showing an ultradian rhythm. The height of the black bars represents the proportion of time spent active within a 30 min window. Grey shading represents night.

The majority of individual activity periods followed an ultradian rhythm (*n* = 19) with a mean period length of 6.71 ± 1.43 h (figure 1*a–c*). The remaining individuals (*n* = 4) showed an approximate daily rhythm of 24.14 ± 0.34 h. Individuals were active on average for 11.4 + 1.9 h per day across a mean of 4.9 ± 1.0 episodic activity bouts (figure 1*c*). The mean bout duration was 138.7 ± 101.5 min across all activity bouts (*n* = 1357 bouts, range 30–630 min). The duration of activity bouts varied greatly between individuals (electronic supplementary material, figure S1 and table S2). Actograms of individual activity are provided in the electronic supplementary material, figure S2.

**Figure 2.**
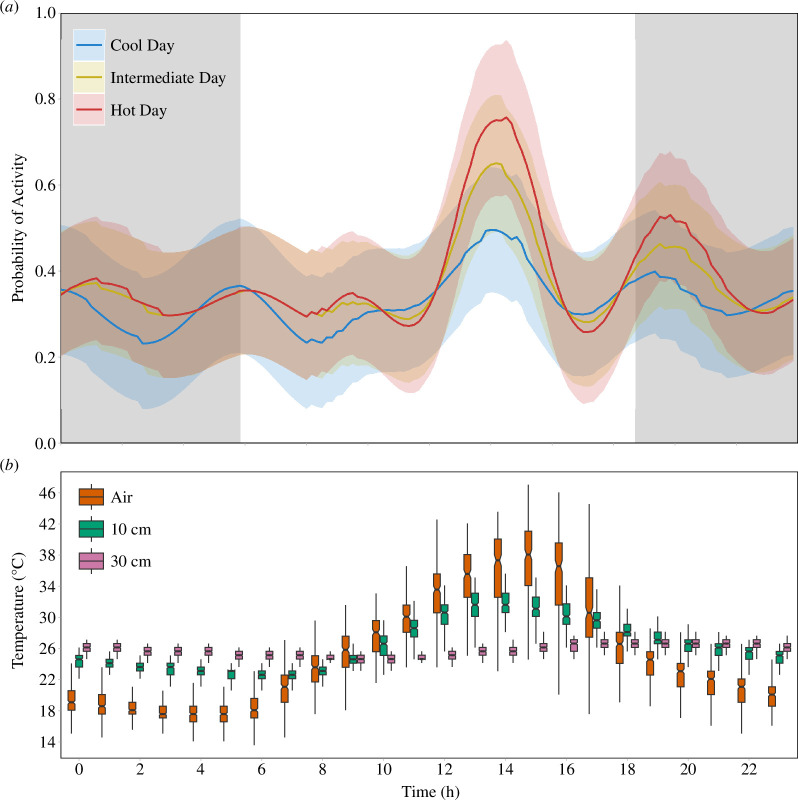
Relationship between activity and temperature in a population of highveld mole-rats. (*a*) The predictions from a mixed model (table 1) predicting overall mole-rat activity on real temperature data from a relatively cool day (blue), a day with intermediate temperatures (yellow) and a relatively hot day (red). Shaded areas in the plot indicate 95% confidence intervals and grey shading indicates the period of darkness. (*b*) Mean hourly temperature at three levels: air temperature (red), soil temperature at 10 cm (green) and soil temperature at 30 cm (purple). Boxes represent the lower and upper quartiles, with a line at the median (notches indicate the 95% CI), and whiskers show the minimum and maximum values (excluding outliers).

The temperature at 10 cm depth predicted the mole-rat activity better than the measurements of the temperature at 30 cm and of ambient temperature (figure 2*a,b*). Including the temperature at 10 cm into these models provided a substantially better fit than including the temperature at 30 cm or ambient temperature (table 1, *r*^2^ = 0.299; 0.227; 0.234, respectively). Higher temperatures were associated with increased mole-rat activity, in particular during the afternoons when mole-rats show their most prominent activity peak (figure 2*b*, table 1, electronic supplementary material, figure S3).

**Table 1.** The effects of temperature on activity patterns of highveld mole-rats. We modelled activity with a four-phasic sinus/cosinus function that breaks the 24 h day into four phases. We built three different models, each for one temperature measurement, and allowed the peaks and the positions of the wave function to vary with the respective temperatures.

	Int.	cos(4t)	sin(**4t**)	temp	cos(**4t**)* temp	sin(**4t**)* temp	AIC	ΔAIC	logLik	marginal ***R***^**2**^	cond. ***R***^**2**^
soil temp (10 cm)											
estimate	−0.124	−0.064	0.223	−0.009	−0.239	0.210	51314.4	0	−25649.4	0.299	0.563
s.e.	0.072	0.015	0.015	0.001	0.015	0.015					
*p*-value	0.083	<0.001	<0.001	<0.001	<0.001	<0.001					
air temp											
estimate	−0.123	−0.042	0.227	0.143	−0.148	0.282	51489.0	174.6	−25736.5	0.234	0.493
s.e.	0.070	0.015	0.015	0.011	0.015	0.015					
*p*-value	0.076	0.004	<0.001	<0.001	<0.001	<0.001					
soil temp (30 cm)											
estimate	−0.136	−0.052	0.220	0.139	−0.274	−0.126	51667.1	352.7	−25 825.6	0.227	0.514
s.e.	0.071	0.015	0.015	0.012	0.015	0.015					
*p*-value	0.057	<0.001	<0.001	<0.001	<0.001	<0.001					

## 4. Discussion

In our study, we employed biologging techniques to investigate the daily activity rhythms in wild highveld mole-rats. Previous laboratory studies have shown that under constant light and temperature conditions, highveld mole-rats have endogenously expressed circadian rhythms in their melatonin secretion, body temperature and locomotor activity which are readily entrained to light and temperature cycles [23,29,33]. Our results show that activity bouts of wild highveld mole-rats follow an ultradian rhythm in the majority of individuals from our study. The mole-rats all displayed 4–5 activity bouts per day, each lasting approximately 2 h. The intensity of these activity bouts was contingent on the temperature at a depth of 10 cm (where mole-rats dig the majority of their foraging tunnels [50]) so that the mole-rats were more active on hotter days and during hotter times of the day.

There are several possible explanations as to why there are differences in activity patterns between wild and captive populations. Chief among those is that the periodicity of activity may be a flexible adaptation to ecological conditions and individual energy expenditure [3,8]. In other mammals with ultradian activity rhythms (e.g. voles), diet and metabolism play a key role in determining the period length between active bouts. Many vole species rely upon hindgut microbial fermentation and consumption of faecal pellets (coprophagy or caecotrophy) to maximize nutrient recovery from their high-fibre, low-protein diet of grasses and forbs [7,52]. As a result, voles must forage until satiated, rest to digest, and forage again 2–3 h later when their digestive tract is empty [6]. Voles may shift from a circadian rhythm to an ultradian rhythm when they are under higher energy demands due to an imbalance between energy expended during foraging and energy gained from diet [8]. Like voles, mole-rats rely upon microbial fermentation and coprophagy to extract nutrients from a similar fibre-rich and energy-poor diet of roots and tubers [20,53–55]. In the wild, mole-rats must expend considerable energy by digging with their teeth in order to locate food [56], whereas in captivity mole-rats are usually fed ad libitum with sweet potato and other energy-rich vegetables. These differences in diet and energy demand may contribute to changes in activity patterns and may also explain some of the large between group and between individual variation of activity in our population. Additionally, captive studies have primarily been conducted on single individuals, and activity rhythms may be disrupted by social cues in complete groups [20,26,40].

Highveld mole-rats were most active during the hottest part of the day and were more active on hotter days than on colder ones, suggesting that the ideal temperature for excavating activity is around 30°C. Although previous studies have found that activity is often linked to temperature in the highveld mole-rat [29] and other African mole-rats [36–40,42], our study suggests that highveld mole-rats do not necessarily avoid heat. In fact, there are compelling reasons why highveld mole-rats might be well adapted to high temperatures. Highveld mole-rats maintain one of the highest body temperatures among mole-rats (over 35°C [57]) and have a narrow thermoneutral zone (TNZ, 30–32°C [57]). These values correspond well to the temperature in the soil at 10 cm depth during peak activity (figure 2). A higher body temperature and TNZ requires high-energy demands to maintain a stable body temperature if the ambient temperature drops below the lower limit of the TNZ. However, these demands can be met by timing activity bouts to burrow temperatures matching their TNZ and body temperature (i.e. behavioural thermoregulation). The metabolic rate of highveld mole-rats (0.68 ml O_2_ g^−1^ h^−1^ [57]) is lower compared with other mole-rats in the genus *Cryptomys*, which may further help to offset the energetic costs of burrowing and allow them to function more effectively in higher temperatures [58–60]. Indeed, it has been suggested that *Cryptomys* mole-rats exhibit short-term plasticity in metabolic rate in response to local conditions [60,61]. Lastly, their thermal conductance of 1.14 suggests efficient heat dissipation if temperatures exceed the optimum [58]. These physiological adaptations of highveld mole-rats are nearly identical to the tropical Ansell’s mole-rat (*Fukomys anselli*, body temperature 33.8–36.1°C, thermoneutral zone 28–32°C, metabolic rate 0.63–0.76 ml O_2_ g^−1^ h^−1^, conductance 0.12–0.144 [62,63]) which was also active midday during the peak in ambient temperature [39]. While the physiological properties of highveld mole-rats have not been extensively studied, it is likely that they are well adapted to cope with activity at temperatures above their maximum thermoneutral zone, allowing them to forage efficiently in their habitat during hot periods.

Four of the mole-rats in this study were predicted to show an activity period length around 24 h. One explanation is that the 24 h rhythm is an artefact of the periodogram analysis. Each of these individuals show less prominent peaks around 6 h on their periodogram (figure 1*b*) and exhibit between four and six activity bouts per day over the course of the study (see electronic supplementary material, table S2). The smaller peaks in the periodogram may indicate that an ultradian rhythm is present, but is statistically overshadowed by a 24 h rhythm. These four individuals came from the two largest groups (seven and 10 animals). It is possible that individuals in larger groups show less synchronization among individuals, leading to greater variation in individual activity rhythms.

## Data Availability

The dataset and R scripts used for analysis and to generate figures are available from the Dryad Digital Repository [64]. Supplementary material is available online [65].
